# Developing Bayesian adaptive methods for estimating sensitivity thresholds (*d*′) in Yes-No and forced-choice tasks

**DOI:** 10.3389/fpsyg.2015.01070

**Published:** 2015-08-04

**Authors:** Luis A. Lesmes, Zhong-Lin Lu, Jongsoo Baek, Nina Tran, Barbara A. Dosher, Thomas D. Albright

**Affiliations:** ^1^Adaptive Sensory Technology, LLCBoston, MA, USA; ^2^Schepens Eye Research Institute, Massachusetts Eye and Ear InfirmaryBoston, MA, USA; ^3^Vision Center Laboratory, Department of Biology, Salk Institute for Biological Studies, University of California, San DiegoSan Diego, CA, USA; ^4^Laboratory of Brain Processes, Ohio State UniversityColumbus, OH, USA; ^5^Memory, Attention, and Perception Laboratory, University of California, IrvineIrvine, CA, USA

**Keywords:** signal detection, adaptive psychophysics, Yes-No, forced-choice, stimulus placement, rating, cuing, decision criterion

## Abstract

Motivated by Signal Detection Theory (SDT), we developed a family of novel adaptive methods that estimate the sensitivity threshold—the signal intensity corresponding to a pre-defined sensitivity level (*d*′ = 1)—in *Yes-No* (YN) and *Forced-Choice* (FC) detection tasks. Rather than focus stimulus sampling to estimate a single level of *%Yes* or *%Correct*, the current methods sample psychometric functions more broadly, to concurrently estimate sensitivity and decision factors, and thereby estimate thresholds that are independent of decision confounds. Developed for four tasks—(1) *simple YN detection*, (2) *cued YN detection*, which cues the observer's response state before each trial, (3) *rated YN detection*, which incorporates a *Not Sure* response, and (4) *FC detection*—the *qYN* and *qFC* methods yield sensitivity thresholds that are independent of the task's decision structure (*YN* or *FC*) and/or the observer's subjective response state. Results from simulation and psychophysics suggest that 25 trials (and sometimes less) are sufficient to estimate *YN* thresholds with reasonable precision (s.d. = 0.10–0.15 decimal log units), but more trials are needed for *FC* thresholds. When the same subjects were tested across tasks of simple, cued, rated, and FC detection, adaptive threshold estimates exhibited excellent agreement with the method of constant stimuli (MCS), and with each other. These *YN* adaptive methods deliver criterion-free thresholds that have previously been exclusive to *FC* methods.

## Introduction

The measurement of detection thresholds, which characterize the sensitivity of sensory systems simply and concisely, provides the foundation of many perceptual studies. The prevalent methods for measuring detection thresholds are the *Yes-No* (*YN*) and *Forced-Choice* (*FC*) tasks. For the sensory researcher, consideration of whether to use *YN* or *FC* tasks has historically focused on the tradeoff between statistical efficiency and decision criterion dependence.

It's known that the *YN* task is more efficient than the *FC* task; results from simulations and psychophysics have demonstrated that, given the same number of trials, *YN* threshold estimates exhibit approximately 25–50% the variability (i.e., standard deviation) of FC threshold estimates (McKee et al., 1985; King-Smith et al., 1994). The primary statistical advantage of the *YN* task is provided by the wider dynamic range of the YN psychometric function (Leek et al., 2000; Klein, 2001; Leek, 2001; Jakel and Wichmann, 2006). The truncation of the 2IFC psychometric function results in a broad range of low intensities at which observers can guess correctly at a 50% rate. Relative to *FC* tasks, the *YN* task also demonstrates practical advantages: (1) for naïve observers presented with low signal intensities, the *YN* response is often more comfortable than being “forced-to-choose” between multiple intervals or alternatives; (2) stimuli presented in multiple intervals of temporal *FC* tasks interact (Yeshurun et al., 2008); (3) improving the efficiency of *FC* methods by increasing the number of alternatives challenges attention or memory processes (Jakel and Wichmann, 2006). Despite these factors, the notable disadvantage of the *YN* task is the significant contribution of the observer's decision criterion (response bias) to detection behavior. In the view of the psychophysical community, the problem of criterion-dependence (bias-contamination) of *YN* thresholds has out-weighed the *YN* task's efficiency, and most laboratories favor the FC task.

The current study reconsiders the problem of decision criterion dependence in the *YN* task. We resolve the issue by developing adaptive methods that combine elements of Signal Detection Theory (SDT) and Bayesian adaptive inference to concurrently estimate sensitivity and decision parameters. For simple *YN* detection, and more elaborate *YN* tasks that cue the observer's response state (cued detection) or incorporate a rated response *(rated detection*), our new methods deliver threshold estimates that are independent of decision criteria that vary with subjective response state or task structure (*YN* or *FC*). These methods maintain the efficiency advantages of the *YN* task, but deliver criterion-free thresholds that have been the presumed domain of *FC* methods.

### Existing adaptive methods

In simple *YN* detection, an observer responds *Yes* or *No* to signify the presence or absence of a target signal. The empirical psychometric function, Ψ _*YN*_(*c*), reflects the probability of a *Yes* response as a function of signal intensity. In two-interval forced-choice (*2IFC*) detection, an observer reports which of two intervals contains the target signal. The empirical psychometric function, Ψ _*FC*_ (*c*), reflects the probability of reporting the correct interval, as a function of signal intensity. In both tasks, experiments often characterize observers not by their full psychometric functions, but by a single empirical threshold: the signal intensity corresponding to a pre-defined performance level (e.g., signal intensity, *c*, for which Ψ _*YN*_ (*c*) = 50%, or Ψ _*FC*_ (*c*) = 75%).

Adaptive psychophysical methods use an observer's responses to focus stimulus presentation to pre-defined regions of the empirical psychometric function (Treutwein, 1995; Leek, 2001). Their efficiency for estimating empirical thresholds, using non-parametric and parametric approaches to stimulus selection and threshold estimation, has made them indispensable for collecting psychophysical data in the lab and clinic. Several dozen adaptive methods have been developed for the *FC* task (Treutwein, 1995; Leek, 2001; Lu and Dosher, 2013). Notable *FC* methods that apply Bayesian adaptive inference include the *QUEST* method (Watson and Pelli, 1983), and the Ψ method (Kontsevich and Tyler, 1999); the Ψ method concurrently estimates threshold and steepness of the psychometric function using an adaptive algorithm that minimizes the expected entropy (uncertainty) about psychometric parameters.

For the *YN* task, existing adaptive methods typically target an empirical threshold—the signal intensity corresponding to a specific *Yes* rate in the middle range of the psychometric function (Kaernbach, 1990; Green, 1993; Linschoten et al., 2001). Because these methods do not estimate the false alarm rate, they cannot de-confound the effects of decision criterion on *YN* empirical thresholds (Klein, 2001). Thus, *FC* adaptive methods have been the primary mode of data collection in the psychophysical community; their historical advantage is based on the idea that only *FC* methods can yield threshold estimates that are independent of decision criterion (however, see Klein, 2001 and Yeshurun et al., 2008; for more critical views of the *FC* task's criterion-free assumptions).

An alternative approach is suggested by the sensitivity threshold, τ_*d*′_—the signal intensity corresponding to a pre-defined level of sensitivity (e.g., *d*′ = 1). An adaptive method that estimates sensitivity thresholds would provide a concise evaluation of detection behavior independently of the task's decision structure or the observer's subjective response state (Green and Swets, 1966; Wickens, 2002; Macmillan and Creelman, 2005). The prospective problem faced by a sensitivity-based *YN* method is that, without *a priori* knowledge of the false alarm rate (decision criterion), the experimenter does not know the empirical *Yes* rate that corresponds to the sensitivity threshold. Therefore, rather than target a single location on the psychometric function, an adaptive strategy that estimates sensitivity thresholds must sample both the psychometric function's rising function (to estimate sensitivity) and its lower asymptote (to estimate decision criteria). Methods that estimate both sensitivity and decision parameters can distinguish between observers who respond *Yes* to low signal intensities due to low thresholds and those that respond due to high false alarm rates (liberal decision criteria).

### The current study

SDT has motivated thousands of perceptual studies in vision and audition (Swets, 1988), but, to our knowledge, there do not exist adaptive methods that directly estimate sensory thresholds based on sensitivity (*d*′). To address this shortcoming, we develop, test, and validate a family of sensitivity-based Bayesian adaptive methods, which consists of the (1) *quick Yes-No (qYN)*, (2) *quick Yes-No Cuing (qYNC)*, (3) *quick Yes-No Rating (qYNR)*, and (4) *quick Forced-Choice (qFC)* methods.

After introducing the foundation of these methods—the application of SDT to describe detection behavior and Bayesian adaptive inference to estimate SDT parameters—we present validation results from simulation and psychophysical studies. Simulations suggest that as few as 25 trials (and sometimes less) are sufficient to estimate sensitivity thresholds at *d*′ = 1 in YN tasks, with acceptable precision (s.d. = 0.1–0.15 decimal log units). In a psychophysical experiment, the method of constant stimuli (MCS) provided independent validation and the four methods cross-validated each other. When measured in the same subjects across different detection tasks, thresholds estimated at a pre-defined sensitivity level (*d*′ = 1) exhibited excellent agreement (mean differences <0.5 dB, where 1 dB = 0.1 decimal log units). By estimating sensitivity thresholds, these methods resolve the problem that criterion-dependence has presented for previous *YN* adaptive methods.

## Quick yes-no (qYN) and quick forced-choice (qFC) methods

Recent studies have extended Bayesian adaptive methods beyond the single psychometric function, to estimate more complex psychophysical models (Kujala and Lukka, 2006; Lesmes et al., 2006, 2010; Vul et al., 2010; Lu and Dosher, 2013). First, a psychophysical function is defined and parameterized by a functional form with a small number of parameters. This function is translated into performance probability (e.g., probability correct or probability yes). On a trial-to-trial basis, the stimulus selection algorithm evaluates potential stimuli for their expected improvement of model parameter estimates, based on the experimental results obtained thus far. These model-based adaptive methods improve psychophysical testing efficiency and model parameter estimation by leveraging information acquired from each trial with *a priori* knowledge about the model's general functional form.

In this study, we combine Bayesian adaptive inference with a signal detection model framework, which proposes independent contributions of sensory and decision processes to detection behavior. An advantage of this approach is a concise description of simple detection that is easily adapted to more elaborate detection tasks. For example, in cued detection (Gu and Green, 1994), an observer alternates between liberal and conservative response states based on instructions. In rated detection, observers maintain multiple response criteria to respond either *Yes, No*, or *Not Sure* (Watson et al., 1973). For 2IFC detection, observers compare two stimulus intervals and report which one contains signal. The proposition that sensitivity is invariant across detection tasks provides the foundation for testing and validating novel *YN* adaptive methods that deliver threshold estimates that are independent of task (*YN* or *FC*) and/or the observer's subjective response state (liberal or conservative).

The primary focus of the current study is developing adaptive methods to estimate sensitivity thresholds for *YN* tasks, but we also introduce a novel *FC* procedure, the *quick FC* (*qFC*) method. The *qYN* and *qFC* methods presented here estimate sensitivity and decision parameters in four detection tasks: (1) simple *YN* detection, (2) cued *YN* detection, (3) rated *YN* detection, and (4) 2IFC detection. The components of these adaptive methods are standard (Watson and Pelli, 1983; Cobo-Lewis, 1997; Kontsevich and Tyler, 1999), but the novel combination of an *SDT* framework with Bayesian adaptive inference provides rapid sensitivity threshold estimates that are free of decision criterion confounds. A more comprehensive evaluation of the *qFC* method, and its performance relative to previously developed *FC* adaptive methods, is saved for a companion paper. In the current study, the *qFC* will provide a critical demonstration that sensitivity threshold estimates obtained in a *FC* task match those obtained in *YN* tasks.

### Modeling empirical psychometric functions

#### The sensitivity psychometric function (*d*′)

**Figure 1:**
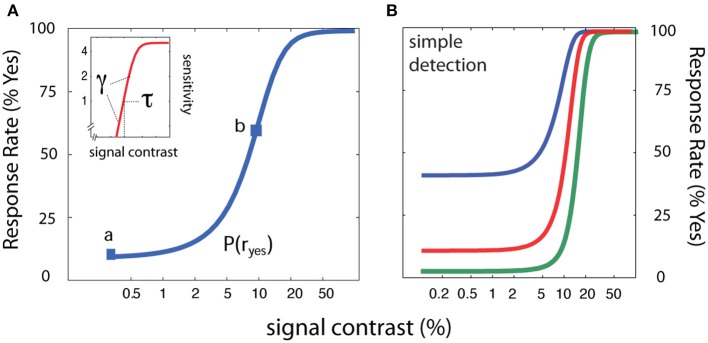
**Psychometric functions for simple YN detection. (A)** In simple detection, the empirical psychometric function (blue) describes the probability of a *Yes* response as a function of signal intensity. A *z-score* transformation of the empirical psychometric function (Equation 1) yields sensitivity (*d*′) as a function of signal intensity (inset; red). **(B)** Three empirical psychometric functions are defined by the same sensitivity function and three decision criteria that correspond to response states with false alarm rates of 2.5, 10, and 40%.

To parameterize the *d*′ psychometric function (Figure 1, inset), we apply a divisive gain-control function of stimulus intensity (e.g., signal contrast), *c*:

(1) $$d^{\prime }(c)=\frac{\beta c^{\gamma }}{\sqrt{\alpha +c^{2\gamma }}},$$

where α is a normalization constant, γ defines the steepness of the *d*′ psychometric function, and β is the saturating function's upper asymptote. This functional form is related to that of the contrast transducer function studied in visual psychophysics (Sperling and Sondhi, 1968; Foley and Legge, 1981) and neurophysiology (Albrecht and Geisler, 1991; Heeger, 1994; Geisler and Albrecht, 1997), and is supported by extensive work on observer models (Lu and Dosher, 2008) (In Appendix A, this parameterization is further justified by comparing its fits of a large simple detection dataset with other contrast transducer functions from the literature). Equation (1) can be re-arranged to yield:

(2) $$d^{\prime }(c)=\frac{\beta {\left(c/\tau \right)}^{\gamma }}{\sqrt{(\beta^{2}-1)+{\left(c/\tau \right)}^{2\gamma }}},$$

where τ is defined to be the sensitivity threshold (signal contrast corresponding to *d*′ = 1). This *d*′ psychometric function is translatable on log abcisssa, as signal contrast is transformed to threshold units: *c*/τ. When plotted on log-log axes, this function is approximately linear over low to medium contrasts and saturates at high contrasts. To simplify the current methods (and because it's practically difficult to reliably distinguish performance at high sensitivity levels; e.g., *d*′ > 4), the asymptote parameter is fixed at 5.0. This simplifying assumption leaves two free parameters to describe the *d*′ psychometric function: the threshold, τ, and the steepness parameter, γ. (This assumption is also justified in Appendix A).

#### Decision criteria

The *d*′ psychometric function, sensitivity as a function of stimulus intensity, is related to the empirical psychometric function, Ψ _*yes*_ (c), which describes the probability of responding *Yes* as a function of stimulus intensity, *c*, by a *z*-score transformation (Green and Swets, 1966):

(3) $$d^{\prime }(c)=z(\Psi_{yes}(c))-z(\Psi_{yes}(0)),$$

where Ψ _*yes*_ (0) is the false alarm rate (Yes rate to null signal). The sensitivity psychometric function, often known as the contrast transducer function, can be defined by a threshold parameter—intensity at which *d*′ = 1—and a steepness parameter (contrast exponent in Equation 2). The inverse relation of Equation (3) describes how a decision criterion parameter translates the *d*′ psychometric function to the empirical *YN* psychometric function (Gu and Green, 1994; Klein, 2001):

(4) $$\Psi_{yes}(c)=1-G\left(\lambda -d^{\prime }(c)\right),$$

where *G*(*x*) is the standard cumulative Gaussian function, and λ = *z*(1−Ψ _*yes*_ (0)) is the decision criterion corresponding to the false alarm rate Ψ _*yes*_ (0). With these assumptions, the empirical *YN* psychometric function for simple detection can be described by three parameters: (1) the threshold of the sensitivity function, τ, (2) the steepness of the sensitivity function, γ, and (3) the decision criterion parameter, λ.

The foundation of the *SDT* model that underlies the *qYN* and *qFC* methods is the proposition that the *d*′ psychometric function is invariant while decision variables change with the observer's response state (i.e., liberal or conservative) or the task's decision structure. In the current study, we apply the basic *SDT* framework to describe detection behavior across four related tasks: (1) simple detection; (2) cued detection, in which the observer alternates between liberal and conservative response states, based on a pre-trial cue; (3) rated detection, in which the observer responds *Yes, No*, or *Not Sure*, and; (4) and two-interval forced-choice detection. The *SDT* model provides a concise description of detection behavior across tasks, which in turn allows efficient Bayesian adaptive estimation of sensitivity and decision parameters.

#### Simple detection (the qYN method)

**Figure 2:**
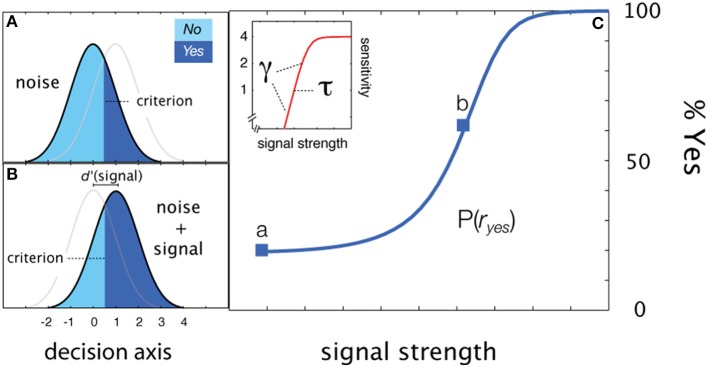
**Simple detection. (A,B)** Distributions of internal representations of signal-present and signal-absent trials. An observer presented only with noise responds *Yes* at a false alarm rate which depends on the criterion. Increasing the signal to a strength corresponding to sensitivity threshold (*d*′ = 1), the observer responds *Yes* at a higher rate: one standard unit (*z*-score) above the false alarm rate. **(C)** Two empirical Yes rates (marked a,b in **C**) signify stimulus levels corresponding to *d*′ = 1 and *d*′ = 0.

For simple detection (see Figure 2), the empirical psychometric function describes the probability of responding *Yes* as a function of stimulus intensity, *c*. In an *SDT* formulation of the psychometric function for simple *YN* detection (Figure 2C), the *Yes* rate is jointly determined by sensitivity for varying contrasts and a decision criterion. Internal sensory events are modeled with standard normal distributions (Figures 2A,B), whose displacement as a function of signal strength is determined by the sensitivity psychometric function (Figure 2C; inset). For a given signal intensity, the corresponding Yes rate, is computed by integrating the area under the signal distribution to the right of the decision criterion. The empirical and sensitivity psychometric functions are therefore directly related through a simple *z*-score transformation. Figures 2A,B demonstrate how sensitivity and decision parameters produce detection behavior at two signal intensities. The empirical psychometric function can be expressed as a function of the *d*′ psychometric function and decision criterion, λ (See Equation 4). The simple *qYN* method estimates three parameters: (1) the threshold of the sensitivity function, τ; (2) the steepness of the sensitivity function, γ, and (3) the decision criterion parameter, λ, that defines the false alarm rate.

#### Cued detection (the qYNC method)

Simple detection can be elaborated for a task that cues observers to alternate between liberal and conservative response states. The observer, presented with a cue before each trial (e.g., directions to be *lax* or *strict*), sets their criterion to effectively sample one of two psychometric functions defined by the sensitivity function and a decision criterion specific to each state:

(5) $$\Psi_{yes}(c,\lambda_{lax})=1-G\left(\lambda_{lax}-d^{\prime }(c)\right),$$

(6) $$\Psi_{yes}(c,\lambda_{strict})=1-G\left(\lambda_{strict}-d^{\prime }(c)\right).$$

By definition, the observer is more likely to respond *Yes* in the liberal state and therefore, in standard units, λ*_lax_* < λ_*strict*_. Due to these order constraints, rather than directly estimate λ _*lax*_ and λ _*strict*_, the *qYNC* method estimates λ _*strict*_ and Δ λ, where:

(7) $$\lambda_{lax}=\lambda_{strict}-\Delta \lambda .$$

The *qYNC* method thus estimates four parameters: (1) the threshold of the sensitivity function, τ; (2) the steepness of the sensitivity function, γ, and (3) two decision criterion parameters λ_*srict*_, and Δλ.

#### Rated detection (the qYNR method)

**Figure 3:**
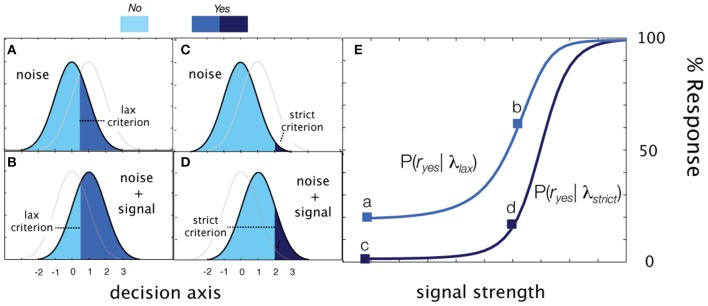
**Cued detection. (A–D)** Detection behavior (Yes rate) generated by two decision criteria in signal-present and signal-absent conditions in cued detection. **(A)** Presented with only noise in the liberal response state, the observer responds with a false alarm rate represented by the area, 1-Φ(λ*_lax_*) **(B)** In the same liberal state, a signal at sensitivity threshold (*d*′ = 1) elicits Yes responses with probability 1-Φ(λ*_lax_*−1. **(C)** When presented with noise in a conservative state, the observer responds with a lower false alarm rate. 1-Φ(λ_*strict*_). **(D)** Likewise, the observer is less likely to say Yes when presented with a signal contrast at the sensitivity threshold. **(E)** Empirical psychometric functions generated by two decision criteria.

**Figure 4:**
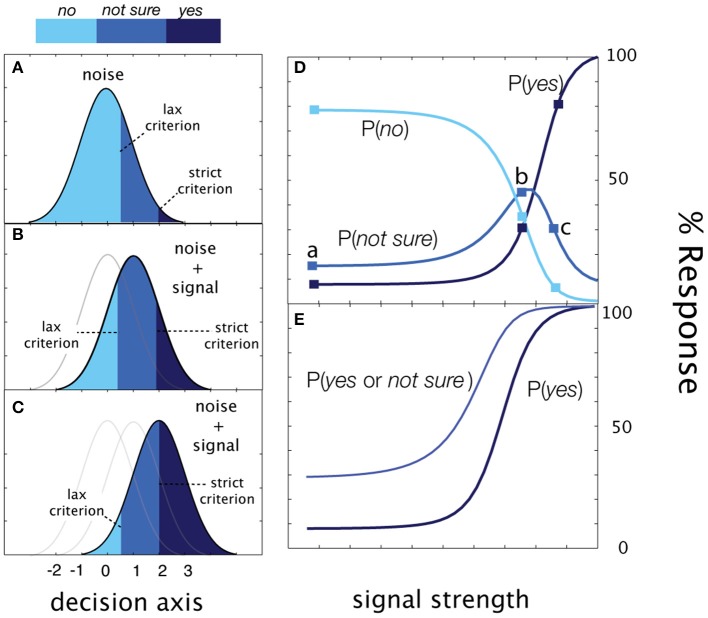
**Rated detection. (A)** Presented with noise alone, the observer responds No with probability, Φ (λ _*lax*_), Yes with probability = 1-Φ (λ _*strict*_), and Not Sure with probability, Φ (λ _*strict*_)-Φ (λ _*lax*_). **(B,C)** As signal intensity (and sensitivity) increases, the relative proportions of the responses change, but sum to 1.0 at each signal intensity. **(D)** The Yes, No, and Not Sure psychometric functions. **(E)** The correspondence between psychometric functions measured in cued and rated detection is apparent when Yes and Not Sure responses are collapsed (c.f., Figure 3E above).

In the simplest version of rated detection, an observer makes one of three detection responses: Yes, No, or Not Sure. **Figure 4** demonstrate how sensitivity and decision criteria produce detection behavior in a yes/no rating experiment. Presented with noise alone, the Yes and No responses depend on a single criterion and Not Sure responses depend on both criteria. As signal intensity (and sensitivity) increases, the relative proportions of the responses change, but sum to 1.0 at each signal intensity. The Yes and No psychometric functions are monotonic functions of signal intensity. The correspondence between psychometric functions measured in cued and rated detection is apparent when Yes and Not Sure responses are collapsed (c.f., Figure 3E). As in cued detection, multiple response criteria generate a pair of psychometric functions; unlike cued detection, which alternates between response states across trials, both liberal and conservative response states (defined by strict and lax criteria) are maintained simultaneously on each trial. For each stimulus intensity *c*, the three response probabilities are:

(8) $$\Psi_{yes}(c,\lambda_{lax},\lambda_{strict})=1-G\left(\lambda_{strict}-d^{\prime }(c)\right),$$

(9) $$\Psi_{not\_sure}(c,\lambda_{lax},\lambda_{strict})=G\left(\lambda_{strict}-d^{\prime }(c)\right)-G\left(\lambda_{lax}-d^{\prime }(c)\right),$$

(10) $$\Psi_{no}(c,\lambda_{lax},\lambda_{strict})=G\left(\lambda_{lax}-d^{\prime }(c)\right).$$

These equations define three psychometric functions that sum to 1.0 at each signal intensity level (see Figure 4). The two empirical psychometric functions observed in cued detection (defined by strict and lax decision criteria) are represented in rated detection, by (1) *Yes* response probability as a function of signal contrast, and (2) collapsing (adding) the *Yes* and *Not Sure* response probabilities as a function of stimulus intensity. Unlike the *qYNC*, the stimulus search for the *qYNR* is one-dimensional over stimulus intensity and responses are ternary.

#### Forced-choice detection (the qFC method)

**Figure 5:**
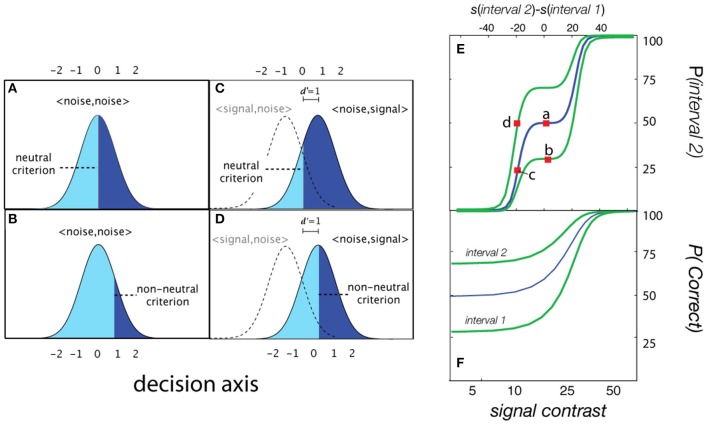
**Forced-choice detection**. When presented with noise in both intervals (*d*′ = 0), the probability of responding *Interval 2* is defined by a **(A)** neutral or **(B)** non-neutral decision criterion. **(C,D)** At higher levels of signal intensity, the probability of responding *Interval 2* is determined by the decision criterion and the integral under the signal distributions corresponding to <noise, signal>. **(E)** The probability of responding *Interval 2* as a function of the relative intensity in the two intervals (*Interval 2 contrast*—*Interval 1 contrast*). **(F)** Empirical psychometric functions for observers with three different interval biases.

For two-interval *FC* detection, the internal sensory representations represent the difference distributions of the sensory activations when the signal is presented either in the first interval, *<signal, noise>*, or in the second interval: *<noise, signal>*. The assumption that FC detection thresholds are criterion-free depends on the observer adopting a neutral criterion (Figure 5A); that is, when the observer is equally likely to respond Interval 1 and Interval 2 with no signal, when *d*′ = 0. For the typical two-interval FC psychometric function (% *Correct* as a function of signal intensity) this assumption results in a lower asymptote of 50%: the two-interval guessing rate (Figure 5F; blue line). With a neutral criterion, the performance level corresponding to the *d*′ = 1 sensitivity threshold is ~76% correct in each interval (Figure 5C), and the psychometric function for each interval is the same (blue line; Figure 5F). Importantly, introduction of an interval bias (non-neutral criterion) results in different empirical psychometric functions for each interval (Figure 5F). Consequently, detection performance in the biased interval is overestimated; given the same level of sensitivity, %Correct in the biased interval is greater than that in the anti-biased interval, or intervals with a neutral criterion. For example, in the biased interval, *%Correct* at *d*′ = 0 is higher than the typical guessing rate (50%). Correspondingly, the empirical thresholds (e.g., 75% *Correct*) in the two intervals are different. An alternative “unwrapped” representation of the empirical psychometric function (Figure 5E) represents the probability of responding *Interval 2* as a function of the relative intensity in the two intervals (*Interval 2 contrast*—*Interval 1 contrast*).

The typical two-interval forced-choice psychometric function, Ψ_2*IFC*_, defined as *% Correct* as a function of intensity, is often usefully re-formulated (Klein, 2001) to describe the probability of responding *Interval 2* as a function of the intensity between intervals:

(11) $$\Psi_{interval2}(\vec{c})=1-G\left(\frac{\lambda_{FC}-[d^{\prime }(c_{interval1})-d^{\prime }(c_{interval2})]}{\sqrt{2}}\right),$$

where $\vec{c}=(c_{interval1},c_{interval2})$ describes the stimulus intensities presented in each interval (see Figure 5). The range of the transformed psychometric function, Ψ _*interval*2_, is 0–100%, in contrast to that of Ψ*_2IFC_*: 50–100%. It's assumed that the **d*′* psychometric function for each interval is the same for the YN task; an additional factor of $1/\sqrt{2}$ converts *d*′ to empirical response rate (Green and Swets, 1966; Macmillan and Creelman, 2005). With a neutral criterion (λ*_FC_* = 0), the probability of responding *Interval 2* when neither interval contains signal is 50%. In this case, the *% Correct* psychometric functions for each interval are the same.

With a non-neutral decision criterion, the Ψ_2IFC_ psychometric function does not pass through the *50%* response rate for the null stimulus condition (i.e., when both intervals contain noise). Because the *d*′ function is a nonlinear function of intensity, when is presented on linear axes of relative intensity (as in Figure 5), it does not look like the cumulative standard normal function: the function flattens out (or pinches) in regions near *d*′ = 0. The degree of apparent pinching depends both on the sensitivity threshold and how rapidly **d*′* changes in the low sensitivity region, which is determined by the steepness of the **d*′* psychometric function.

In addition to two sensitivity parameters, the *qFC* method estimates the decision parameter, λ _*FC*_. The stimulus search is defined over a single dimension of relative stimulus intensity (see Figure 5E). Therefore, unlike existing *FC* adaptive methods, the *qFC* method selects both the signal intensity and *the interval in which the signal is presented.*

#### Lapse errors

The value of the psychometric function described in Equation (4) (also see Equations 5–11) range from the *false alarm rate* to 100%; however, lapses in the observer's behavior (e.g., inattention or finger errors) prevent measurement of detection behavior over this full range (Swanson and Birch, 1992; Green, 1995; Wichmann and Hill, 2001a). In the present case, we assume that observers make such errors at a rate of ε = 2% and that *Yes* or *No* responses are distributed equally on such trials. With this lapse rate, the upper asymptote of the empirical psychometric function is 99%. Therefore, the parameterization of the empirical psychometric functions is:

(12) $$\Psi^{\prime }(c)=\varepsilon +(1-\varepsilon )\Psi (c).$$

Including this lapse factor accurately reflects the contribution of non-sensory factors (inattention, blinks, finger errors) that produce unexpected responses (at the lowest and highest stimulus intensities) with some low probability (<5%).

### Bayesian adaptive parameter estimation

Following the landmark application of Bayesian adaptive inference to estimate FC thresholds (QUEST; Watson and Pelli, 1983), King-Smith et al. (1994) applied it to measure YN thresholds with an assumed false alarm rate. For *FC* tasks, more complicated Bayesian adaptive methods that estimate the threshold and steepness of the psychometric function have been developed (Cobo-Lewis, 1996; King-Smith and Rose, 1997; Snoeren and Puts, 1997; Kontsevich and Tyler, 1999; Remus and Collins, 2007). Relative to QUEST or staircase procedures that target a single threshold on the psychometric function, these methods sample multiple foci on the psychometric function. For example, to estimate the threshold and steepness of the 2AFC psychometric function, the Ψ method (Kontsevich and Tyler, 1999) samples stimuli from the threshold region (*75% Correct*), in addition to two loci (approximately *70* and *90% Correct)* that facilitate estimation of the steepness of the psychometric function.

The *qYN* and *qFC* methods combine the principles of SDT and Bayesian adaptive inference to rapidly estimate sensitivity and bias parameters that describe detection behavior in *YN* and *FC* tasks. These methods increase testing efficiency by improving stimulus sampling, via an adaptive algorithm that improves the gain of information (i.e., the decrease of entropy) over a multi-dimensional space of SDT parameters. Before the experiment begins, a probability density function, defined over sensitivity and bias parameters, represents prior knowledge of the observer's detection behavior. During the experiment, stimulus selection incorporates a one-step ahead search algorithm, to evaluate the next trial's potential outcomes over a space of potential stimuli, and thereby find stimuli that improve information gained about SDT parameters. Following each trial, a Bayesian update refines the SDT parameter estimates given that trial's outcomes.

Taken together, these features provide efficient stimulus sampling that leverages information acquired during the experiment with *a priori* knowledge about the model's general functional form and priors over its parameters. This process greatly accelerates the gain of information about detection behavior, via estimates of sensitivity and decision parameters. As a result, these methods rapidly yield sensitivity threshold estimates that are free of decision-level confounds.

The *qYN* and *qFC* methods share four components that allow quick, efficient, estimation of the psychometric functions: (1) an *SDT* model that characterizes empirical psychometric functions for detection with sensitivity and decision parameters, (2) an adaptive algorithm for stimulus selection that uses a one-step-ahead search to gain information about SDT parameters, (3) Bayesian inference update of the parameter estimates following each trial, and (4) a stop rule.

#### Parameter and stimulus spaces

To estimate psychophysical functions using Bayesian adaptive inference, it's necessary to define a parameter space that comprises candidate psychophysical functions and select stimuli that discriminate between candidate functions, based on updating knowledge. The *qYN* and *qFC* methods estimate three parameters, and the *qYNC* and *qYNR* methods estimate four parameters (see Figures 2–5). Cued and rated detection tasks generate two empirical psychometric functions because they reflect additional criterion states, but requires only a single additional decision parameter, relative to simple detection, which already includes one criterion. With the exception of the *qYNC* method, the stimulus search space is one-dimensional over stimulus intensity; for the *qYNC*, the stimulus search algorithm calculates over the stimulus intensity dimension for each cued response state (liberal or conservative).

#### Priors

A probability density function, *p*(θ), is defined over the model parameter space that defines detection behavior. Before any data are collected (trial *t* = 0) the initial prior distribution, *p*_*t* = 0_ (θ), represents foreknowledge of model parameters. The use of priors is a basic advantage of applying Bayesian methods (Kuss et al., 2005), which can usefully influence the testing strategy, based on existing test results and/or demographics (Turpin et al., 2007). Bayesian inference is used to update the prior *pdf* (and its corresponding parameter estimates), given the subject's responses to stimuli presented during the experiment. Due to the well-known difficulty of estimating the steepness of the psychometric function, its measurement requires a large investment in data collection. For this reason, the priors embody the most restrictive information about the steepness parameter, but are much less strict about decision criterion or psychometric threshold relative to previous methods. Previous Bayesian methods have applied strict priors for parameters that define psychometric steepness (Watson and Pelli, 1983), and/or false alarm rate (King-Smith et al., 1994). Strict priors, which constrain model fits and increase method precision, leave these methods vulnerable to biases due to mismatches between the model priors and the underlying parameters in a particular testing situation (Alcalá-Quintana and García-Pérez, 2004). The great reduction in data collection that strict priors provide is generally an acceptable tradeoff for these potential biases.

#### Bayesian update

For each adaptive method, a gridded parameter space θ, either three- (τ, γ, λ) or four- (τ, γ, λ _*strict*_, Δ λ) dimensional, represents the full range of empirical psychometric functions that potentially describe the observer. The asymptote (β) and lapse rate (ε) parameters are set a priori.

Before the experiment, an initial prior *p*_*t* = 0_(θ) that represents *a priori* knowledge of the observer's psychometric functions is defined. During the experiment, Bayesian inference is used to update the prior distribution, *p*(θ), to the posterior distribution, *p*_*t*_ (θ |s,r), following each trial *t* based on the observer's response *r*_*t*_ to that trial's stimulus, *s*_*t*_.

Rather than consider responses as correct vs. incorrect, the current methods consider the probabilities of responding *Yes* or *No* (*qYN* and *qYNC*), *Yes, No*, or *Not Sure* (*qYNR*), or *Interval 1* or *Interval 2* (*qFC*). Stimulus conditions are defined by the possible levels of stimulus intensity in *qYN, qYNR*, and *qFC*, and the combination of stimulus intensity and response state in *qYNC*. Following standard practice, the probability of responding *r*_*t*_ given stimulus *s*_*t*_ is estimated by weighing empirical response rates by the prior:

(13) $$p_{t}(r_{t}|s_{t})=\sum_{\theta }\Psi^{\prime }(r_{t},s_{t})p_{t}(\theta ).$$

This normalization factor, sometimes called the *probability of the data*, is used to estimate the posterior pdf, *p*_*t*_ (θ |*s*,*r*), via *Bayes Rule*:

(14) $$p_{t}(\theta |s,r)=\frac{p_{t}(\theta )\Psi^{\prime }(r,s)}{p_{t}(r|s)}.$$

#### Stimulus selection

In the current study, we applied a stimulus selection algorithm that simulates the possible outcomes of the next trial to select stimuli that maximally (or nearly maximally) improve estimates of model parameters. Stimulus selection is calculated using a one-step-ahead search and a criterion of minimum expected entropy (Cobo-Lewis, 1996; Kontsevich and Tyler, 1999; Kujala and Lukka, 2006) or equivalently, maximum expected information gain (Kujala and Lukka, 2006). By simulating the possible outcomes of the next trial for each possible stimulus, and evaluating stimuli for their expected effects on the prior (as it's updated to posterior), stimulus selection avoids the least informative regions of the stimulus space that lead to inefficiency in pre-determined sampling schemes.

Before each trial, the next trial is simulated, and the above analysis (Equations 13 and 14) is completed for all the possible responses to all the possible experimental stimuli. For the next trial, the stimulus is the one that minimizes the expectation of the posterior entropy following the next trial (Cobo-Lewis, 1996; Kontsevich and Tyler, 1999; Kujala and Lukka, 2006). After calculating the simulated posterior, *p*_*p* + 1_(θ|*s*,*r*), for all the possible responses to each possible stimulus, the entropies of the simulated posteriors are calculated:

(15) $$H_{t\;+\;1}(s,r)=-\sum_{\theta }p_{t\;+\;1}(\theta |s,r)\log(p_{t\;+\;1}(\theta |s,r)).$$

Expected entropy is then calculated as a function of possible stimuli by weighing posterior entropies by response probabilities:

(16) $$E[H_{t\;+\;1}(s)]=\sum_{r}H_{t\;+\;1}(s,r)p_{t}(r|s).$$

For trial *t* + 1 the stimulus condition providing the lowest expected entropy is chosen:

(17) $${\vec{s}}_{t\;+\;1}=\arg\underset{s}{\min}E[H_{t\;+\;1}(s)].$$

By presenting the observer with the stimulus condition providing the minimum expected entropy for *p*_*t* + 1_(θ), the current methods obtain the most information about the observer's psychometric functions. Although the current application defines the optimal stimulus relative to minimum expected entropy, other applications might use other strategies, such as minimum expected variance.

#### Re-iteration and stop rules

After the observer finishes trial *t* + 1, the posterior corresponding to *s*_*t* + 1_ and the observed response *r* is saved and used as the prior distribution for the subsequent trial:

(18) $$p_{t\;+\;1}(\theta )=p_{t}(\theta |s,r)$$

The *qYN* and *qFC* methods terminate either when the total number of trials reaches a pre-specified value (as implemented in this paper) or when the precision of the threshold estimate reaches a pre-determined level (Alcala-Quintana and Garcia-Perez, 2005; Tanner, 2008).

## Simulation studies

### Demonstration of qYN in simple detection

Movie 1 demonstrates the *qYN* applied to measure the sensitivity threshold in simple *YN* detection. The simulated observer (see Figure 1B) exhibits the contrast threshold = 10.0%, contrast exponent = 2.0, and decision criterion = 1.28 (false alarm rate = 10%). The executable MATLAB programs used to generate Movie 1 are available for download (http://lobes.osu.edu/qYN.php). Interested readers can adjust the parameters of the simulated observer as well as the priors of the qYN parameters in the simulation program.

In this demonstration, the *qYN* method's parameter space is a three-dimensional grid; the parameter ranges are: 0.1–99% for the threshold of the sensitivity function, 0.25–12 for its steepness parameter, and −0.5–3 standard (*z*) units for decision criterion. The decision parameters correspond to false alarm rates that range from <1–75%. The one-dimensional stimulus space ranges from 0.1 to 99% contrast, with 0.25 dB sampling resolution.

The initial prior was the normalized product of one-dimensional marginals (Kuss et al., 2005), which are log-symmetric around the modes of the sensitivity parameters (τ = 1%, γ = 2) and linear-symmetric for the decision criterion parameter (in *z*-space). The prior probabilities are weakly informative—widely spread over a range of each parameter values—to avoid the risk of mismatched priors. The priors used in the current demo represent a compromise that allows flexibility for estimating a wide range of false alarm rates, without sacrifice of precision.

The trial sequence in Movie 1 demonstrates how the *qYN* method efficiently samples the dynamic region of the *YN* psychometric function: the range of signal contrasts from 5 to 20%. The simulation demonstrates that the sensitivity threshold estimate, defined by a flat marginal prior before the experiment's start, rapidly takes shape in the region of the true threshold value (10% contrast). By trial 10, the pdf develops mass near the value of the true sensitivity threshold. As the experiment evolves, the refinement of the sensitivity threshold estimate is demonstrated by change in the width of the confidence intervals, as a function of trial number. Throughout the experiment, the information gain function exhibits several local maxima that correspond to stimulus regions informing sensitivity and decision parameters; namely, (1) the threshold region, (2) the lowest intensity stimulus, which informs the decision criterion; and (3, 4) lower and higher signal intensities that flank the threshold region and inform the psychometric steepness parameter (Kontsevich and Tyler, 1999).

### Simulation of qYN in simple detection

#### Method

To evaluate the qYN in simple detection, we simulated an observer in three different response states. With the same underlying sensitivity parameters (contrast threshold = 10%; contrast exponent = 2), each response state generated a different empirical psychometric function (false alarm rates = 2.5, 10, and 40%; see Figure 1B). To evaluate the accuracy and precision of threshold estimates obtained with the *qYN*, the simulation was repeated for 1000 iterations.

The three-dimensional parameter space, which represents all possible psychometric functions, consists of *d*′ threshold (τ) from 0.0025 to 1.25 (59 samples on a logarithmic scale), *d*′ slope (γ) from 0.4 to 10 (58 samples on a logarithmic scale), and bias (λ) from −1 to 3 standard deviation (56 samples in linear space). The prior probability distribution was set to a broadly distributed hyperbolic secant, centered on initial guess over each parameter. Possible contrasts are from 0.001 to 0.99 with 120 equally spaced samples on a logarithmic scale. The true parameters of the simulated observer were τ = 10%, γ = 2, and λ = 1.

#### Results

Simulations were used to evaluate the *qYN* method's accuracy and precision for estimating sensitivity thresholds and to examine the method's stimulus sampling patterns.

##### Stimulus sampling

**Figure 6:**
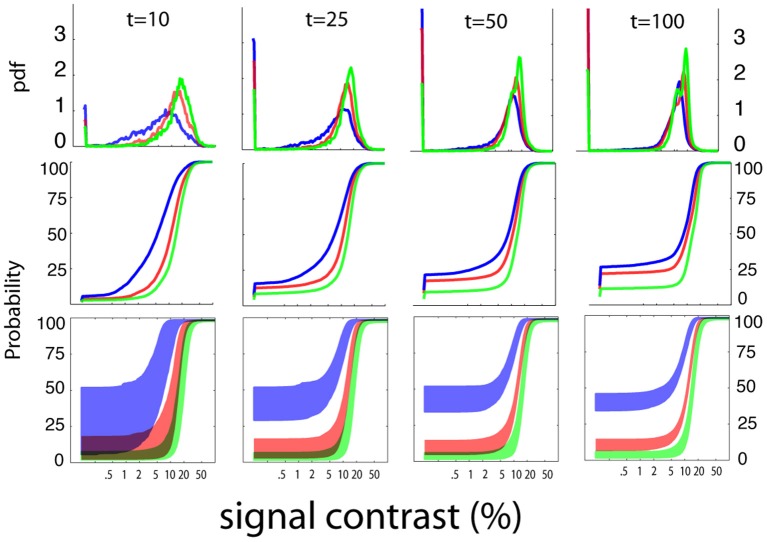
**Stimulus sampling**. The aggregate patterns of stimulus sampling and psychometric function estimates are presented, for stopping points of 10, 25, 50, and 100 qYN trials, in different columns. To characterize the qYN's stimulus sampling, ordinary histograms (upper row), and cumulative histograms (middle row), are presented as a function of signal contrast. Within 10 trials, sampling is focused to signal contrasts near the sensitivity threshold (10%). As testing progresses, most of the stimulus sampling coincides with the dynamic region of the empirical psychometric function. The sampling pattern also critically depends on the observer's decision criterion.

Figure 6 summarizes the pattern of the *qYN*'s Bayesian stimulus sampling, by presenting ordinary and cumulative histograms of the stimuli presented over the aggregate of 1000 simulation iterations. The *qYN*'s implicit sampling strategy (Kujala and Lukka, 2006), which gains information about underlying SDT model parameters, differs from prior methods that explicitly target a single location (e.g., the 50% empirical threshold) on the psychometric function. Instead, the *qYN* alternates sampling between several regions that provide information about sensitivity and decision parameters. Over the first part of the experiment (~10 trials), stimulus placement focuses on estimating threshold; the expected information gain function shows a global maximum near the true threshold. However, sampling does not solely depend on the sensitivity threshold, but also on the decision criterion; when observers respond liberally (high false alarm rate), more stimuli are presented at lower intensities, to distinguish whether *Yes* responses at low intensities signify a low threshold or a liberal response state. By trial 10, the most liberally responding observer (Figure 6, green line; false alarm rate = 40%) evokes sampling at predominantly lower intensities. After the first 10 trials (see Movie 1), the expected information gain function exhibits local maxima reflecting the lowest stimulus intensities (to refine false alarm rate estimates), and contrast levels above and below the threshold (to estimate psychometric steepness).

In addition to increased sampling of low intensities for liberally-responding observers, the cumulative stimulus histograms (Figure 6; middle row) demonstrate correspondence between the steepest portions of the stimulus sampling distributions and the dynamic regions of the psychometric function. The result of this broad sampling strategy is an efficient resolution of different regions of the psychometric function: both the lower asymptote (defined primarily by decision parameters) and the dynamic region (defined by sensitivity parameters). This sampling pattern is critical for distinguishing between observers who respond *Yes* to low intensities due to low thresholds and those with high false alarm rates. The convergence of psychometric function estimates is demonstrated by their decreasing variability with increasing trial number (bottom row). The shaded regions represent the variability of function estimates via the interquartile ranges of predicted response probabilities, as a function of signal contrast. The psychometric function estimates are generally accurate (but imprecise) in early testing, and then narrow significantly with increasing trial number.

In most traditional adaptive procedures such as the staircase and QUEST procedures, stimulus selection focuses on the region near the estimated threshold. This could result in inter-trial dependency: successive presentation of a narrow range of stimulus levels could change observer's underlying sensitivity and bias. In contrast, qYN uses a wide range of stimulus levels in order to estimate both sensitivity and bias. The risk of inter-trial dependency is minimized.

##### Threshold estimation: accuracy and precision

**Figure 7:**
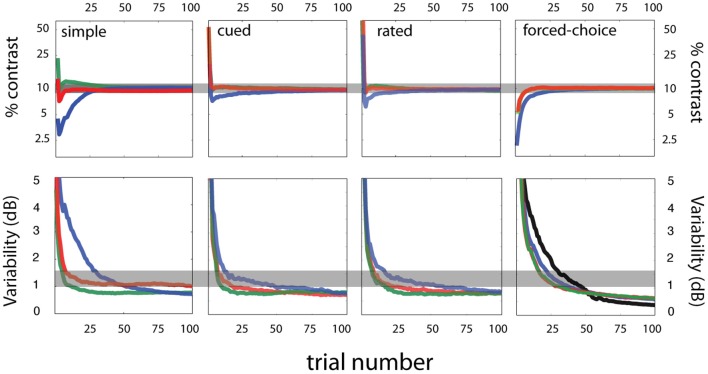
**Simulation results**. Sensitivity threshold estimates—accuracy and variability. Accuracy (top row) and variability (bottom row) of sensitivity threshold estimates are presented as a function of trial number for four tasks—simple, cued, rated, and forced-choice detection (arranged by column). For each task, three response states (red, blue, green) were simulated for an observer with the same sensitivity parameters (contrast threshold = 10%; contrast exponent = 2; see **Figure 10** for more details on the simulated response states). Across the different criterion states simulated for each task, the general willingness to respond is signified by color: conservative (green), intermediate (red), and liberal (blue) response states.

Figure 7 (first column) presents the accuracy and precision of sensitivity threshold estimates obtained with the *qYN* method, as a function of trial number. Accuracy is characterized by how threshold estimates deviate from the true threshold value (top row), and precision is characterized by the standard deviation of threshold estimates (bottom row). The bias (in dB) in threshold estimates was calculated as:

(19) $${\hat{\tau }}_{bias}=10log10\left(\frac{\hat{\tau }}{\tau_{true}}\right).$$

Figure 7 (top row) demonstrates that threshold estimates rapidly converge for response states that vary in decision criterion. Over the range of false alarm rates presently tested, threshold bias decreases below 0.5 dB by 25 trials, (1 dB = 0.10 log units). The threshold precision is represented by the standard deviation of threshold estimates (Figure 7, bottom row). Within 25 trials, the *qYN* delivers threshold estimates with 0.1 log unit precision, for false alarm rates of 2.5 and 10%. The lower threshold precision (~1.5 dB) observed with the high false alarm rate (40%) is a pattern consistent with that exhibited by *FC* methods with relatively high guessing rates (King-Smith et al., 1994).

The confounding effects of decision criterion on empirical *Yes* rates are apparent in the evolution of sensitivity threshold estimates (Figure 7; upper left panel). For the most liberally responding observer, initial threshold estimates undershoot the true value: during this testing stage the *YN* method must distinguish a low threshold from a liberal decision criterion (high false alarm rate). Thus, threshold estimates are initially low, until additional data collection identifies the high false alarm rate and resolves the proper sensitivity threshold. In this way, the *qYN* distinguishes between observers with relatively conservative and liberal response states.

##### Effects of the prior

**Figure 8:**
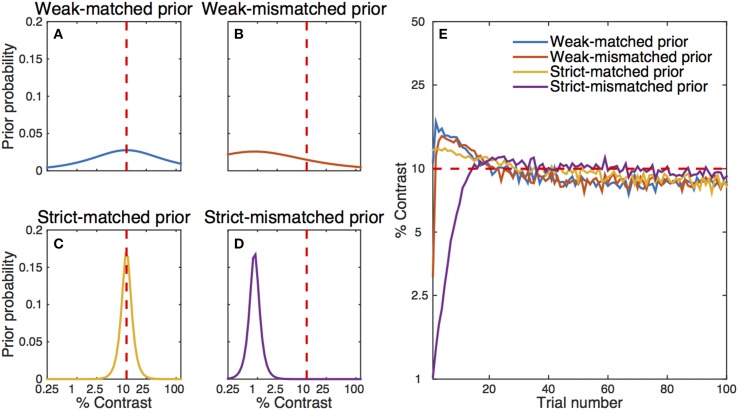
**Simulation results with different initial prior distributions: (A) a weak- matched prior, (B) a weak-mismatched prior, (C) a strict-matched prior, and (D) a strict-mismatched prior**. The red dashed line represents the simulated observer's true parameter. **(E)** Estimated contrast threshold as a function of trial number. The simulation results start with estimates *after* the simulation of the first trial, whereas the left panels represent prior distributions *before* the simulation of the first trial. The qYN/qFC methods gain much information in the first few trials, so that even a single (first) trial changes the posterior distribution. Note that there is some residual bias in the estimated contrast threshold even after 300 iterations of the procedure.

Often, the prior probabilities are assumed to be uniform over a range of values. Alternatively, it is possible to use prior knowledge to focus more narrowly on likely values of the parameters. In our simple qYN simulation, the prior distribution was spread over a broad range of parameter values. It is well known that a more informative prior distribution could change the starting point of parameter estimation and make the estimation process even faster. To illustrate this effect, we conducted another set of simulations with four different prior settings for contrast threshold (Figures 8A–D): (1) a weak matched prior (prior mode = 10%, prior confidence = 1.6), (2) a weak mismatched prior (mode = 0.9%, confidence = 1.6), (3) a strict matched prior (mode = 10%, confidence = 11.5), and (4) a strict mismatched prior (mode = 0.9%, confidence = 11.5). Broadly distributed priors were set for contrast exponent (mode = 2.0, confidence = 6.1) and decision criterion (mode = 1, confidence = 2.1) in all simulations. The average estimated contrast threshold from 300 iterations of the simple qYN procedure is shown as a function of trial number in Figure 8E. Results show that the two simple qYN procedures with weakly informative priors—either matched or mismatched—generated essentially the same performance after a few trials. A strict matched prior can enhance the performance of the procedure, but there is a risk of getting deteriorated accuracy when the informative prior is mismatched. It is important to note that bias caused by a mismatched prior can be overcome after testing dozens of trials (e.g., 20 trials in this simulation). In practice, the prior for the qYN procedure can be informed by prior knowledge or pilot data.

Effects of prior on the steepness parameter are not simulated in this investigation. The prior used on the steepness parameter is not strict, especially in the contrast exponent domain. The analysis of the data collected with the MCS in Appendix A is instructive, because it demonstrates that (1) fixing the steepness parameter at 2.0 did not have an appreciable effect on fitting a large simple detection dataset, and (2) the estimated steepness parameter ranged from 1.8 to 2.2 across observers, with bootstrap variability estimates ranging from 0.3 to 0.5 for individual observers. Based on these results, we feel that setting the range of the contrast exponent between 1 and 4 is not strict. Furthermore, because the target performance level of the contrast threshold is usually placed around the steepest part of the psychometric function (e.g., *d*′ = 1), a mismatch of the steepness prior has very little effect on threshold estimates. It's also important to note that QUEST assumes a fixed steepness parameter.

##### Parameter space, stimulus space, and computing time

**Figure 9:**
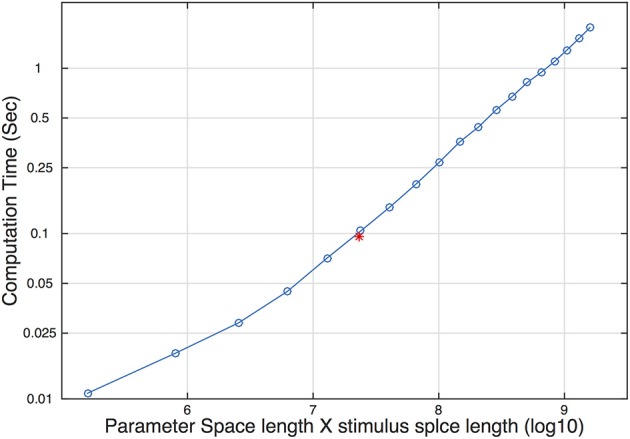
**Computation time as a function of the size of the parameter and stimulus space**. The red asterisk represents the size and the corresponding computation time used in our simulation study.

In each trial, the qYN procedure updates the posterior distribution and selects the most informative stimulus level for the next trial. Such steps require internal computations of a huge 59 × 58 × 56 × 120 array. The size of the array affects the speed of pre- and post-trial computations. To determine the limitation caused by array size on real psychophysical experiments, we measured computation time as a function of array size, determined by the size of the parameter and stimulus space. The computation time increased linearly with the size of the array (Figure 9). The red asterisk represents the size of the array and its computation time used in our simulation. On a Core 2 Duo laptop, the computation time was around 100 ms. Considering that the typical inter-trial interval is > 200 ms in most psychophysical experiments, our choice of parameter and stimulus space is acceptable in practice. We expect that rapid advances in computer technology would eliminate the practical limitation.

### Simulations of qYN in cued, rated, and forced-choice detection

#### Method

**Figure 10:**
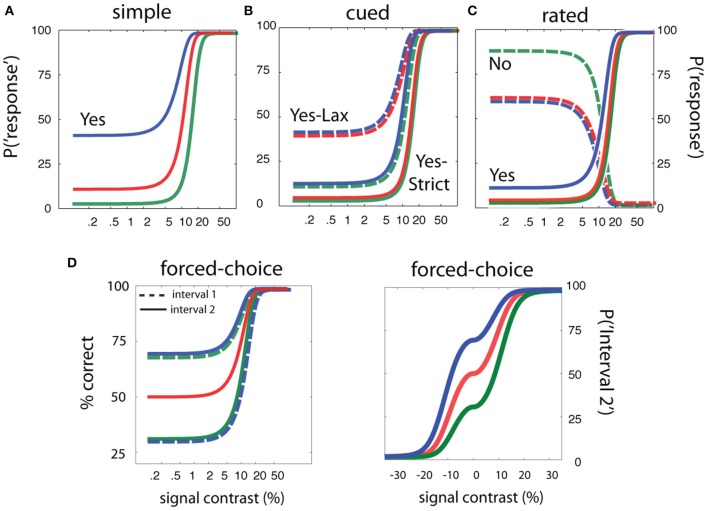
**Simulation design for sensitivity-based adaptive methods**. The simulations of adaptive methods for simple, cued, rated, and forced-choice detection assume sensitivity parameters (threshold = 10% and contrast exponent = 2) are invariant across a range of detection tasks and response states. **(A)** Psychometric functions in simple detection with false alarm rates of 2.5, 10, and 40%. **(B)** Psychometric functions in cued detection with three pairs of false alarm rates for the conservative and liberal states: (2.5, 10%), (2.5, 40%), (10, 40%). **(C)** Psychometric functions in rated detection with three pairs of decision criteria that produce *Yes* and *No* response rates of 2.5 and 90% (green), 2.5 and 60% (red), and 10 and 90% (blue) in signal-absent trials. **(D)** Psychometric function in two-interval forced-choice detection with a bias toward responding Interval 2 (red), a bias toward responding interval 1 (green), or no bias (blue).

To complement the results obtained for simple detection, and demonstrate that sensitivity-based adaptive methods can yield threshold estimates that are independent of task and the subject's decision state, we used simulations to evaluate methods developed for cued, rated, and FC detection. For each of the cued, rated, and FC detection tasks, we simulated an observer with the same sensitivity parameters specified in the previous *qYN* simulations (contrast threshold = 10%, contrast exponent = 2), in three different decision states representing distinct criteria. Figure 10 demonstrates the broad range of simulated detection behavior, given invariant sensitivity parameters and decision criteria that vary with task and response state. Demonstration movies of each of the cued, rated, and forced-detection methods are presented in Movies 2–4 respectively.

The psychometric functions obtained in cued and rated detection depend on two decision criteria. In cued detection, a cue directs the observer to operate either under lax or strict decision criteria that define liberal and conservative response states. For rated detection, the observer maintains multiple response states on each trial, and can thus respond “Not Sure,” in addition to Yes and No. In some rating YN experiments, researchers have used four or more response categories. Choosing a large number of response categories, thus more parameters to be estimated, increases the number of required trials to obtain reliable measurements as well as computational resource. Since the primary interest is often on obtaining sensitivity threshold independent of decision criteria, qYNR uses rating procedure with only three states. For cued and rated detection simulations, we used decision parameters corresponding to the three response states simulated for simple detection.

For evaluation of the *qYNC* method applied to cued detection, the simulated decision states comprised conservative and liberal response states with corresponding false alarm rates: (i) 2.5 and 10%, (ii) 2.5 and 40%, and (iii) 10 and 40%. The overall willingness to respond *Yes* increases across these three decision states. In rated detection, the decision criteria that define psychometric functions for *Yes, No*, and *Not sure* are directly related to those in cued detection (see Figure 4). For simulations of the *qYNR* method, the same criteria simulated for cued detection were used; these criteria respectively define *Yes* and *No* response rates to the null stimulus: 2.5 and 90%, 2.5 and 60%, and 10 and 60%.

For FC detection, it's commonly presumed that empirical thresholds (defined by *% Correct*) are criterion-free. However, both naïve and experienced observers can exhibit interval or alternative biases that undermine this assumption (Klein, 2001; Jakel and Wichmann, 2006). To address this issue, we developed the *quick FC* method, which estimates the decision criterion (interval bias) for two-interval *FC* detection. This distinguishes the *qFC* method from previous methods (QUEST, Watson and Pelli, 1983; the Ψ method, Kontsevich and Tyler, 1999) that have exclusively focused on estimating empirical thresholds, and therefore do not provide clear ways to detect, characterize, and estimate parameters for biased observers. The *qFC* method may be especially useful for application to naive human or animal observers who may adopt non-neutral decision strategies before they gain psychophysical experience (Jakel and Wichmann, 2006). Given the same sensitivity parameters simulated in the *YN* tasks, we simulated response states with (i) first-interval, (ii) second-interval, and (iii) neutral interval bias. For general comparison, we simulated the application of QUEST (Watson and Pelli, 1983) to measure empirical thresholds (at 75% correct) for an un-biased observer.

#### Results

Despite the range of simulated detection behavior (Figure 10), which varied in decision structure and response states, the sensitivity threshold estimates obtained with the *qYN* and *qFC* methods exhibit consistent patterns. Across a range of response states in simple, rated, cued, and FC detection tasks, the variability of threshold estimates starts to decrease consistently by 10–15 trials (Figure 7; bottom row). By trial 25, bias decreases below 0.5 dB and variability below 1.5 dB for all response states (shaded region marks true threshold ±0.5 dB). As reported in previous studies, and also seen for the qYN in simple detection, (1) the convergence of precise threshold estimates is more rapid in YN tasks, relative to FC tasks, and (2) in YN tasks, threshold convergence is most rapid for relatively conservative response states (green). In simple, cued, and rated detection, response states corresponding to lower guessing rates (Yes rates to null stimuli) yield threshold estimates with lower variability. In those cases, only 15–20 trials are needed for sufficient accuracy (bias < 0.5 dB) and precision (~1 dB) of sensitivity threshold estimates. The importance of psychometric functions with an expanded dynamic range is supported by the observation that the precision of YN threshold estimates with 40% false alarm rate (first column, lower row, green line) is similar to that observed for FC threshold estimates (last column, second row).

**Figure 11:**
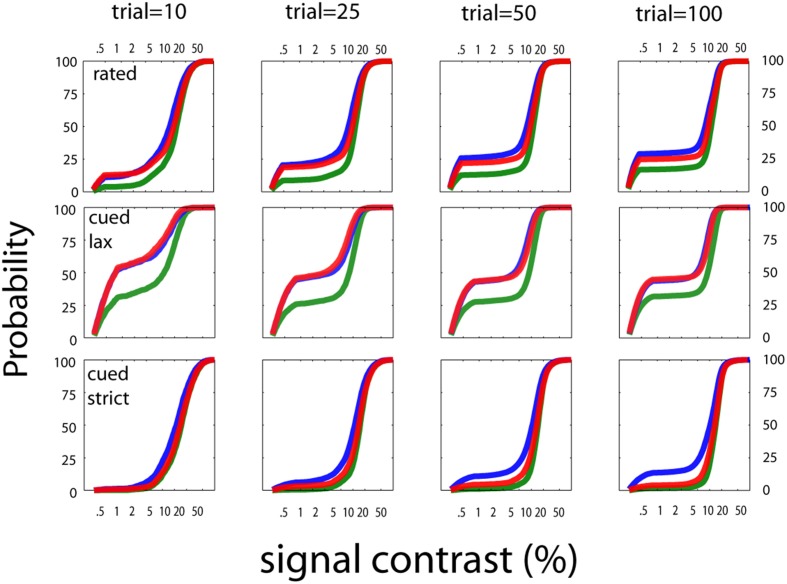
**Stimulus sampling patterns for cued and rated detection**. The stimulus sampling patterns of the *qYNC* and *qYNR* methods are presented for stopping points of 10, 25, 50, and 100 completed trials. The general pattern is similar to that demonstrated by the quick YN method; namely, that stimulus sampling is (1) mostly focused to the dynamic region(s) the psychometric function(s), and (2) increased at the lowest-intensity stimuli for liberal response states. This sampling strategy allows the concurrent estimation of sensitivity and decision parameters in an efficient way.

**Figure 12:**
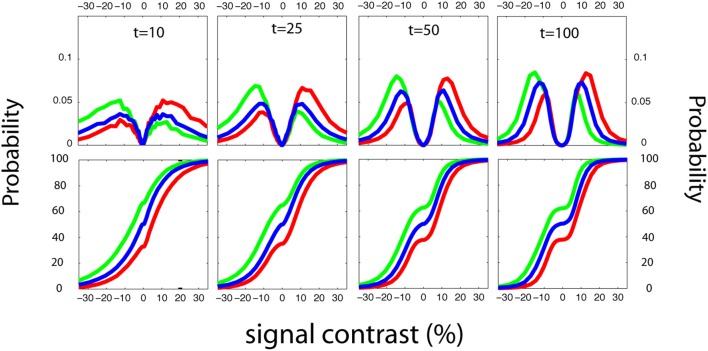
**Stimulus sampling patterns for forced-choice detection**. The *quick FC*'s sampling is demonstrated by ordinary and cumulative stimulus histograms. The *qFC* method demonstrates a pattern similar those demonstrated by the YN adaptive methods; namely, that the stimulus sampling pattern matches the empirical psychometric function. For example, fewer stimuli are presented in the second interval to an observer who is biased against the second interval (Figure 5). This asymmetry is evident as early as the 10th trial.

The stimulus sampling patterns of the *qYNC* and *qYNR* methods are presented for stopping points of 10, 25, 50, and 100 completed trials in Figure 11. The general pattern is similar to that demonstrated by the qYN method; namely, that stimulus sampling is (1) mostly focused to the dynamic region(s) the psychometric function(s), and (2) increased at the lowest-intensity stimuli for liberal response states. This sampling strategy allows the concurrent estimation of sensitivity and decision parameters in an efficient way. The behavior of the *qFC* in FC detection was similar to the *qYN* in simple detection when YN false alarm rates were high (40%). In turn, the *qFC* demonstrates better precision (lower variability) than QUEST, over the first 50 trials (see Figure 12; bottom row, far right panel). The *qFC* stimulus sampling demonstrates similar patterns to the *qYN* methods. The adaptive sampling matches stimulus presentation to the dynamic regions of the empirical psychometric function. These simulations suggest that estimating sensitivity thresholds is a promising approach to efficiently characterize detection performance. To evaluate how these simulations translate to real applications, we conducted a psychophysical study.

The stimulus sampling patterns of the qFC method is demonstrated by ordinary and cumulative stimulus histograms (Figure 12). The *qFC* method demonstrates a pattern similar to those demonstrated by the YN adaptive methods; namely, that the stimulus sampling pattern matches the empirical psychometric function. For example, fewer stimuli are presented in the second interval to an observer who is biased against the second interval (Figure 5). This asymmetry is evident as early as the 10th trial.

**Figure 13:**
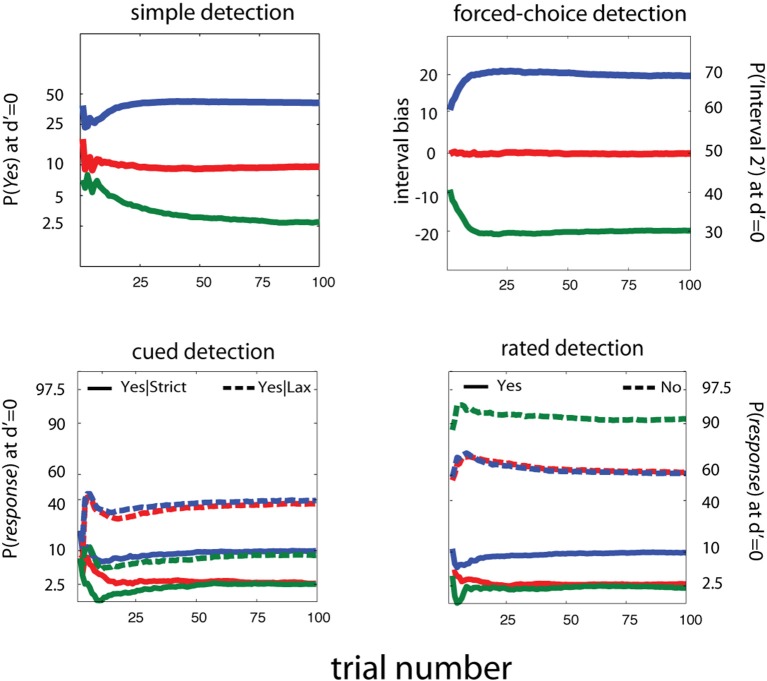
**Estimating Decision Criteria**. For invariance of sensitivity threshold estimates across detection tasks, it's necessary to estimate and account for decision criteria across different response states. Here we present estimates of response probabilities at *d*′ = 0, for simple, cued, rated, and forced-choice detection tasks. It's apparent that, unlike previous adaptive methods developed for YN and FC tasks, these methods can reliably distinguish a broad range of decision-level behaviors. For simple and cued detection, 25 trials are sufficient for the qYN and qYNC methods to distinguish *Yes* response rates that correspond to different response states: 2.5, 10, and 40%. For rated detection, the corresponding response rates are 2.5, 10, and 40, for Yes Responses and 90 and 60% for No responses. Simulation results for the *qFC* are presented for un-biased (red) and interval-biased (green, blue) response states in forced-choice detection (see Figure 4). When presented with a blank stimulus in both intervals, the biased observers responded Interval 2 with probabilities of 70 or 30% (interval bias of ±20%). Similarly to the methods developed for the YN tasks, the *qFC* can reliably distinguish response behavior corresponding to biased or unbiased forced-choice detection.

For invariance of sensitivity threshold estimates across detection tasks, it's necessary to estimate and account for decision criteria across different response states. In Figure 13, we present estimates of response probabilities at *d*′ = 0, for simple, cued, rated, and FC detection tasks. It's apparent that, unlike previous adaptive methods developed for YN and FC tasks, these methods can reliably distinguish a broad range of decision-level behaviors. For simple and cued detection, 25 trials are sufficient for the qYN and qYNC methods to distinguish *Yes* response rates that correspond to different response states: 2.5, 10, and 40%. For rated detection, the corresponding response rates are 2.5, 10, and 40 for Yes Responses and 90 and 60% for No responses. Simulation results for the *qFC* are presented for un-biased (red) and interval-biased (green, blue) response states in FC detection (see Figure 5). When presented with a blank stimulus in both intervals, the biased observers responded Interval 2 with probabilities of 70 or 30% (interval bias of ±20%). Similarly to the methods developed for the YN tasks, the *qFC* can reliably distinguish response behavior corresponding to biased or unbiased FC detection.

## Psychophysical study

In a psychophysical study, the *qYN* and *qFC* adaptive methods were evaluated by independent validation with the *MCS* and cross-validation between the *qYN* and *qFC* methods.

### Method

#### Apparatus

The experiment was conducted on a Windows-compatible computer running *PsychToolbox* extensions (Brainard, 1997; Pelli, 1997). The stimuli were displayed on a Dell 17-inch color CRT monitor updating at a 120 Hz refresh rate. A special circuit changed the display to a monochromatic mode, with high grayscale resolution (14 bits) and luminance levels linearized via a lookup table (Li et al., 2003). Stimuli were viewed binocularly with natural pupil at a viewing distance of approximately 72 cm in dim light.

#### Participants

Three naïve observers (TC, JA, and CH) and one of the authors (JB) participated in the experiment. All observers had corrected-to-normal vision and two (JB, CH) were experienced in psychophysical studies.

#### Stimuli

The signal stimuli were Gaussian-windowed sinusoidal gratings, oriented = +45 degrees from vertical, shown at fovea. The luminance profile of the Gabor stimulus is described by:

(20) $$\begin{array}{l} L(x,y)=L_{0}\{1.0+c\times \sin[2\pi f(x\cos\theta +y\sin\theta )]\;\\ \;\times e^{-(x^{2}+y^{2})/2\sigma^{2}}\}, \end{array}$$

where *c* is the signal contrast, σ = 0.42° is the standard deviation of the Gaussian window, and the background luminance L_0_ was set in the middle of the dynamic range of the display (L_min_ = 3.1 cd/m^2^; L_max_ = 120 cd/m^2^). The signal stimuli were rendered on a 64 × 64 pixel grid, extending 2.08 × 2.08° of visual angle. External noise images were constructed using 2 by 2 pixel elements (0.064 × 0.064°). Each noise element's contrast level was drawn independently from a Gaussian distribution with mean of 0 and standard deviation of 16% contrast. On each trial, noise images were composed of elements with jointly independent, identically distributed contrasts.

#### Design and procedure

Each observer completed four experimental sessions. Each session, devoted to a single detection task, consisted of four adaptive runs (each lasting 100 trials). These trials were inter-mixed with the *MCS*, which was used to collect 50 trials at each of a number of pre-determined signal contrast levels. In each task, the target signal sequence was the same: a fixation-cross presented in the center of the screen for 500 ms was followed by the stimulus sequence. The basic sequence, which consists of three 8.3 ms frames: a noise frame, a signal frame, and another (independent) noise frame, is presented in each task. Each task varied the procedure for presenting this sequence.

##### Simple detection

Following stimulus presentation, observers used a key press to respond *Yes* if they thought the signal was presented and *No* otherwise. No feedback was provided. *MCS* trials were collected at eight signal contrast levels: null contrast and seven levels spaced log-linearly from 8 to 60% contrast (50 trial × 8 signal contrasts = 400 MCS trials).

##### Cued detection

Before the fixation cross was presented on each trial, the observer's response state was cued by one of two words–*Lax* or *Strict*–displayed for 500 ms at the screen's center. In practice trials that preceded the experiment, observers were instructed to be conservative in responding *Yes* on the *Strict* trials and liberal on the *Lax* trials. A payoff structure was implemented to reinforce and maintain the distinction between response states throughout the session. On the *Lax* trials, the observers gained eight points for *hits*, while losing two points for *false alarms*; on the *Strict* trials, observers received two points for *hits* and lost eight points for *false alarms*. No trial-by-trial feedback was given. Instead, during a break every 25 trials, the observer was presented with the running totals of (1) points won and lost for the previous 25 trials and (2) points won and lost for the entire session. *MCS* trials were collected at eight signal contrast levels for both conservative and liberal response states (50 trials × 8 signal contrasts × 2 response states = 800 *MCS* trials). The same contrast levels were used as in simple detection.

##### Rated detection

The stimulus sequence was the same as simple detection, but observers responded *Yes, No*, or *Not sure*, with respect to signal presence. *MCS* trials were collected at eight stimulus levels: (50 trials × 8 signal contrasts = 400 MCS trials). The same contrast levels were used as in simple detection.

##### FC detection

In contrast to the *YN* tasks, *FC* detection presented a stimulus sequence over two intervals separated by 1 s. The target interval contained the signal sequence and the other contained a blank frame presented between two noise frames. Observers used a key press to respond *Interval 1* or *Interval 2*, depending on which interval contained the signal. No feedback was given. *MCS* trials were collected over 11 stimulus conditions. Ten conditions were defined by five contrast levels (spaced log-linearly), which were presented in the first or second interval. The last condition presented the null stimulus (0% contrast) in both intervals: (50 trials × 11 stimulus conditions = 550 trials).

### Results

The *MCS* data were used as baseline to validate the proposition that an invariant sensitivity function accounts for detection behavior across different tasks and/or response states. In addition, we sought to validate that sensitivity threshold estimates obtained with the *MCS* agreed with *qYN* and *qFC* threshold estimates, and that *qYN* and *qFC* threshold estimates agreed with each other.

#### Independent and cross-validation: MCS and qYN

**Figure 14:**
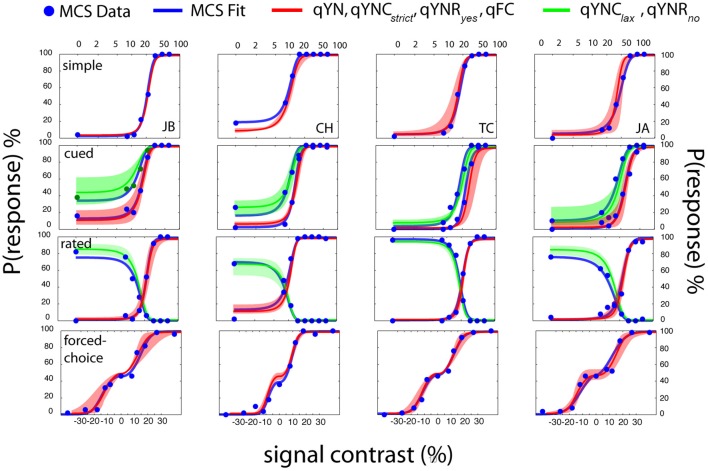
**Psychophysical validation**. Data from each detection task are organized by row and data from each observer is organized by column. Simple and forced-choice detection tasks define one psychometric function, and the cued-criterion and rated detection tasks define two functions. *MCS* data (blue dots) were fit with an SDT model that generated empirical psychometric functions (blue lines) from a task-invariant sensitivity psychometric function and task-specific decision parameters. For each observer in each task, the mean *SDT* parameters (averaged across 4 runs of 100 trials) were used to generate the mean psychometric function estimates (red, green lines) obtained from adaptive methods. The shaded regions (red, green) represent the full range of psychometric function estimates—the minimum and maximum response probabilities obtained across the four adaptive runs, as a function of signal contrast.

Figure 14 presents results obtained with the *MCS* and four adaptive methods (*qYN, qYNC, qYNR*, and *qFC*) in four different detection tasks. Each row presents the data from a single detection task and each column presents data from a single observer. The cued and rated detection tasks each produce two empirical psychometric functions. For the FC task (bottom row) presenting the un-wrapped psychometric function (probability of responding *Interval 2* as a function of relative contrast), rather than the typical psychometric function (*%Correct)*, helps to de-clutter the data presentation. In addition to the raw data collected with the *MCS* (blue dots), psychometric functions obtained from *MCS* model fits (blue lines), and psychometric functions obtained from *qYN* and *qFC* methods are presented (red, green lines). For each task and observer, the psychometric functions were calculated from the sensitivity and decision parameters averaged across the four adaptive runs. The shaded regions represent the range of psychometric function estimates; that is, the lowest and highest estimates of response probabilities obtained across four adaptive runs. A general inspection suggests excellent agreement between psychometric functions obtained with the *MCS* and *qYN* and *qFC* methods, although estimation of sensitivity threshold is the focus of the new adaptive methods.

The *MCS* data clearly demonstrate the importance of characterizing detection behavior using both sensitivity and decision factors. For example, consider observer *JB*'s performance in cued detection (Figure 14, second row-first column). Given the same stimulus (signal contrast = 11%), the conservative and liberal response states produced large *Yes* rate differences: 10 vs. 40%. Furthermore, the change in decision criterion produces an approximately two-fold difference between the *50% Yes* thresholds in the two response states (10 vs. 20% *contrast*).

*MCS* data collected across the four detection tasks were analyzed by testing a basic *SDT* model comprised of a single *d*′ psychometric function and task-specific decision parameters. In addition to two sensitivity parameters—the threshold, τ, and steepness, γ, of the *d*′ function– this model includes six decision criteria: one each for simple and FC detection, and two each for cued and rated detection. A bootstrapχ ^2^ analysis, which assessed goodness-of-fit (see Appendix B for details), concluded that this unified *SDT* model could not be rejected for any of the observers (*p* > 0.5). Figure 14 presents the empirical psychometric functions predicted by the MCS model fits (blue lines). Table 1 (Appendix B) presents sensitivity parameter estimates, (τ_*MCS*_, γ_*MCS*_), obtained for each observer. The bootstrap-estimated variability of *MCS* threshold estimates was low (<0.2 dB), due to the relatively large dataset per subject (>2000 trials across four tasks).

**Table 1:** **Parameter estimates for threshold and steepness of the d-prime psychometric function obtained with the MCS obtained across four detection tasks**.

**Observer**	**Parameters**	
	**${\hat{\tau }}_{MCS}$**	**${\hat{\gamma }}_{MCS}$**
JB	0.153 (0.18)	2.39 (0.38)
CH	0.095 (0.23)	2.06 (0.48)
TC	0.140 (0.19)	2.35 (0.34)
J1	0.144 (0.22)	1.84 (0.31)

Bootstrap generated medians and standard deviations (in dB) are presented.

**Figure 15:**
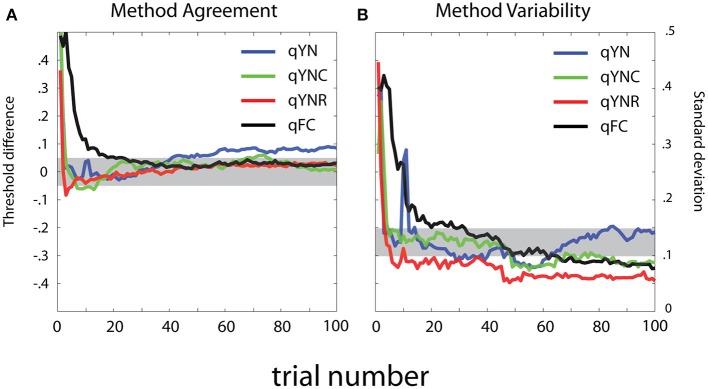
**Method comparison. (A)** Mean and **(B)** standard deviation of log-ratio difference scores are presented as a function of trial number in the adaptive run. **(A)** For method agreement, the shaded region represents excellent agreement (±0.5 dB) between threshold estimates obtained with the *MCS* and the *qYN* and *qFC* adaptive methods. **(B)** For method variability, the shaded region represents standard deviation of difference score estimates that range from 10 to 0.15 log units.

To evaluate method agreement, Figure 15 presents difference scores calculated between sensitivity threshold estimates obtained with the *MCS* and the *qYN* and *qFC* methods. The mean and standard deviation of the difference scores are presented, as a function of trial number in the adaptive run. In general, threshold estimates obtained with the different methods show excellent agreement with the MCS. For the *qYNR* and *qYNC* methods, large differences between thresholds observed with less than 10 trials decrease to <0.5 dB within 20 trials. *qFC* threshold estimates took longer (>30 trials) to approach MCS estimates. The largest threshold differences were demonstrated by the *qYN* method, which exhibited a 0.45 dB difference that persisted up to 100 trials. The apparent increase of the bias of the estimated thresholds with increasing trials number is inconsistent with the simulation results (Figure 7). One possible reason for such discrepancy is that we don't know the true thresholds in the psychophysical study. Although we used the results from the MCS method as the “truth,” the MCS estimates themselves are associated with variabilities and are only approximations of the truth.

The thresholds estimates obtained from the *qYNR* showed excellent precision, with variability decreasing to 1 dB by 15 trials, and to 0.5 dB by 50 trials, whereas the other methods converged more slowly. The variability of *qYN* and *qYNC* threshold estimates decreased below 1.5 dB by trial 25, with the *qYN* showing 1 dB precision. By 60 trials, variability of all four methods reached 1 dB precision. The considerable decrease in threshold variability observed over the earliest epoch of data collection (<10 trials) suggests that the threshold priors do not dominate threshold estimates. Like the residual threshold difference the *qYN* exhibited with 100 trials, another unfavorable aspect of *qYN* behavior is that threshold variability increased from 50 to 100 trials.

### Discussion

For psychophysical validation, we applied a conservative experimental design. To determine the validity of the SDT model that underlies the newly developed *qYN* and *qFC* methods, we examined detection behavior using a more deliberate, classical *MCS*. We concurrently collected data using the MCS to establish some estimate of the “ground truth.” The design was complicated still more by evaluating the task-invariance of sensitivity thresholds. For that reason, participants in our study completed >3000 trials with >2000 MCS trials. Out of the four participants in our experiment, only two had experience in psychophysical experiments: JB was one of the authors and CH was a researcher in our lab. The two other participants (TC and JA) were naïve volunteers with no prior experience as psychophysical subjects before the study. As shown in Figure 14, our methods did not show any degraded efficiency for naive participants. In fact, more precise estimates were obtained from TC than from JB.

These data provided independent evidence that performance across four detection tasks could be accounted for by a single sensitivity psychometric function and task-specific decision criteria. In addition, adaptive methods developed for *YN* and *FC* tasks yielded threshold estimates that agreed with *MCS* estimates. This validation was important because many factors prevent adaptive methods from the idealized performance studied in simulations. A mismatch between the assumed model or assumed parameter priors can result in inaccuracy or imprecision of threshold estimates. These data suggest that the assumption of steepness priors was reasonable. Similarly, non-stationarity of behavior can result in method inefficiency. For example, an observer in a simple detection task may change their decision criterion over the course of an experiment. The psychophysical data obtained with the *qYNR* demonstrated excellent agreement with simulations. As predicted, sensitivity thresholds obtained with as few as 15 trials (for the *qYNR* method), which matched those obtained with the *MCS*, demonstrated excellent precision. Perhaps, relative to other tasks, observers in the rated detection were able to maintain multiple criteria precisely and reliably. Given that 10–20 *YN* trials can be easily completed in 1–2 min, this method shows great potential for rapid, accurate estimates of sensitivity thresholds.

## General conclusion and discussion

### The qYN and qFC methods

The previous development of adaptive methods has focused on targeting pre-defined *%Yes* or *%Correct* performance levels on the empirical psychometric function. Following the development of YN and FC staircase procedures,(von Bekesy, 1947; Wetherill, 1963; Wetherill and Levitt, 1965), the *QUEST* method (Watson and Pelli, 1983) was the landmark application of Bayesian adaptive inference to measure FC thresholds. The Bayesian adaptive approach has since been applied to measure empirical thresholds in YN and FC tasks (Watson and Pelli, 1983; King-Smith et al., 1994; King-Smith and Rose, 1997; Snoeren and Puts, 1997; Alcala-Quintana and Garcia-Perez, 2007; Garcia-Perez and Alcala-Quintana, 2007). In psychophysics, adaptive methods have proved essential in reducing testing times needed for estimating empirical thresholds in *YN* and *FC* tasks.

In the current study, we have developed, tested and validated four Bayesian adaptive methods that estimate sensitivity thresholds in *YN* and *FC* detection tasks. To our knowledge, these are the first adaptive methods that apply the SDT framework to directly estimate sensitivity thresholds. By their previous focus on empirical thresholds, existing adaptive methods have not reliably estimated decision parameters in *YN* and *FC* tasks, but (Kaernbach's 1991, 2001) has attempted to mitigate the contaminating effects of response bias in *YN* and *FC* tasks. Unlike other *YN* methods, (Kaernbach's 1991) staircase method incorporated null stimulus presentation into its adaptive strategy, and adjusted stimulus presentation based on responses to null stimuli. For the FC task, Kaernbach (2001), attempted to diminish the contaminating effects of *FC* response bias by introducing a *Don't Know* response. Though this “unforced-choice” procedure reduces the impact of response biases, it does not estimate any decision parameters that define response bias. Though these methods were the best available approach to mitigating response bias effects on the measurement of empirical thresholds, they fell short of accounting for effects of decision factors on detection behavior. By directly assessing sensitivity and decision parameters, the *qYN* methods resolve the criterion-dependence of *YN* thresholds that has largely prevented its widespread application in psychophysical laboratories. The *qFC* provided a valuable demonstration that sensitivity thresholds obtained in *YN* tasks matched those obtained in the *FC* task, which is the *de-facto* standard for laboratory studies. We believe these novel *YN* methods present strong alternatives to *FC* methods of threshold assessment and hope their availability increases the application of *YN* tasks.

These sensitivity-based methods deliver on an attractive premise of SDT: that detection metrics should not depend on decision-level factors that include the task's decision structure or the observer's response state. This feature is particularly important for comparing measures of perceptual phenomena (e.g., perceptual learning) measured across different experimental designs (Fine and Jacobs, 2002). Application of such tools should be useful in paradigms like subliminal perception, multisensory integration or adaptation, in which it is critical to distinguish the contribution of decision factors to perceptual phenomena.

In this study, we focused on developing quick methods to estimate sensitivity threshold. The objective function used in these methods is minimizing the entropy of the (joint) posterior of the parameters of the full psychometric functions. Given the goal of the methods, the objective function may not be optimal. Alternative objective functions may place differential weights on different parameters of the psychometric function or be based on sensitivity threshold directly.

Development of fast adaptive procedures has been proved to be useful for studying clinical populations. For example, an adaptive procedure for measuring contrast sensitivity function (Lesmes et al., 2010) has been successfully applied to estimate and classify contrast sensitivity functions of patients with amblyopia in very short testing times (Hou et al., 2010). The value of quick adaptive procedures could also be found in many laboratory environments. Naïve observers in psychophysical experiments often fail to respond consistently in long experimental sessions. The short testing time with qYN and qFC would not only reduce the burden on subjects but also enable more reliable estimates observers' performance.

### Future directions for bayesian adaptive methods

Recent development of adaptive methods has focused on methods that estimate more than the empirical threshold of the *FC* psychometric function, like its steepness (King-Smith and Rose, 1997; Snoeren and Puts, 1997; Kontsevich and Tyler, 1999; Kujala and Lukka, 2006; Remus and Collins, 2007) and its upper and lower asymptotes (Cobo-Lewis, 1996; Tanner, 2008).

An emerging generation of adaptive methods exploits computing advances to estimate psychophysical models of increasing complexity (Kujala and Lukka, 2006; Lesmes et al., 2010; Vul et al., 2010). Kujala and Lukka (2006) applied Bayesian adaptive inference to estimate equi-discrimination elliptical contours. The *qYN* and *qFC* methods join the larger family of *quick Methods* that we've developed for rapidly estimating psychological functions. These include the *quick TvC* method, which measures external noise functions (Lesmes et al., 2006), the *quick CSF* method, which measures spatial contrast sensitivity functions (Lesmes et al., 2010), and the quick Surface method, which measures the spatio-temporal contrast sensitivity surface (Lesmes et al., 2009). Given their efficiency, these methods demonstrate great potential for characterizing the perceptual deficits caused by visual neuropathology. Applied to amblyopia, Hou et al. (2010) demonstrated that as few as 50 *quick CSF* trials (<5 min) are needed to characterize the contrast sensitivity function deficits in amblyopia.

The problem addressed by adaptive methods in psychophysics is a general one encountered in many scientific and engineering applications. Given limits in time or other experimental resources (Kujala, 2010), which is the next best observation to make? The convergence of solutions to this problem within psychophysics and across disciplines should help advance the development and application of efficient empirical methods in psychophysics and other domains.

### Conflict of interest statement

Luis A. Lesmes and Zhong-Lin Lu have personal financial interests in Adaptive Sensory Technology, LLC. The other authors declare that the research was conducted in the absence of any commercial or financial relationships that could be construed as a potential conflict of interest.

## Supplementary material

Movie 1 **The *qYN* method applied to estimate the sensitivity threshold in simple detection**. The true YN psychometric function (black line; upper left panel) and the qYN's updating estimate of the psychometric function (red dotted line) are presented. The trial history (upper middle panel) presents the signal contrast presented on each trial and the subsequent response. Each dot represents the contrast of the selected stimulus, as a function of trial number, and the dot's color represents whether the observer responded *Yes* (blue), or *No* (black). Shaded regions that correspond to confidence intervals for the sensitivity threshold estimates (65% in dark red and 95% in light red) as a function of trial number, are overlaid on the trial history. In the adjacent panel (upper right), calculation of the stimulus selection algorithm is presented and the true threshold value is presented for reference (dotted line); expected information gain is normalized by the maximum of the expected information gain estimate. Therefore, the selected stimuli correspond to peaked regions of the information gain function. The Bayesian update of the pdf defined over three *qYN* parameters (threshold and steepness of the sensitivity function and decision criterion) is presented. The one-dimensional marginals for two sensitivity parameters (threshold and steepness) and one decision parameter are presented (lower panels); the ground truth for each parameter is presented for reference (dotted lines).

Movie 2 **The *qYNC* method applied in cued detection**. The upper panels present the true and estimated psychometric functions (left), the trial sequence (middle), and calculation of the pre-trial stimulus selection algorithm (right). The lower panels present sensitivity and decision parameter estimates, via the 2-d marginal pdfs defined over (1) sensitivity parameters (threshold and steepness), and (2) decision criteria: the strict criterion, and the *z*-score difference between the strict and lax criteria. The empirical psychometric functions generated by the liberal and conservative response states are presented for the true observer (blue) and for qYNC estimates (red). In the simulated trial sequence, the contrast of the selected stimulus and the cued response state are presented as a function of trial number. For each trial, the dot's position on the abscissa represents the trial number and the position on the ordinate represents the selected contrast. The outline color signifies the cued response state (red-strict; green-lax) and the dot's filled color signifies the observer's response (blue-Yes; black-No). The shaded regions represent the 65% (dark red) and 95% (lighter red) confidence intervals for sensitivity threshold estimates, as a function of trial number. The peak of the information gain function for the conservative response state is shifted to higher signal contrasts, relative to that of the liberal state. In addition, at lower signal intensities, the stimulus selection function falls off less slowly for the liberal state. The trial sequence demonstrates the *qYNC* method systematically samples different stimulus regions for the liberal and conservative response states. As a result of this stimulus selection pattern, the quick *YNC* is able to sample the dynamic regions of both empirical psychometric functions, and thereby sample a broader range of detection behavior than is possible in simple detection.

Movie 3 **The *qYNR* method applied in rated detection**. The upper panels present the true and estimated psychometric functions (left), the trial sequence (middle), and calculation of the pre-trial stimulus selection algorithm (right). The lower panels represent sensitivity and decision parameter estimates, via the 2-d marginal pdfs defined over (1) sensitivity parameters (threshold and steepness), and (2) decision criteria: the strict criterion, and the *z*-score difference between the strict and lax criteria. In the upper left panel, the true empirical psychometric functions (*Yes* and *No;* blue) are presented with updating *qYNR* estimates (red). In the trial sequence, the signal contrast selected for each trial is represented by the dot's position on the ordinate, and the trial's outcome (*Yes, No*, and *Not Sure* responses) is represented by dot's color (red, blue, and green). The shaded regions represent the 65% (dark red) and 95% (lighter red) confidence intervals for sensitivity threshold estimates, as a function of trial number.

Movie 4 **The *qFC* method applied in forced-choice detection**. The movie demonstrates the quick FC method applied in a two-interval forced-choice detection task. The simulated observer demonstrates a response bias that favors the first interval (60% *Interval 1* vs. 40% *Interval 2* for the null stimulus). The upper panels present the true and estimated psychometric functions (left), the trial sequence (middle), and the pre-trial calculation of the stimulus selection algorithm (right). In addition to the *%Correct* psychometric functions presented for each interval, we also present the unwrapped psychometric function (inset), which describes probability of reporting *Interval 2* as a function of relative contrast in the two intervals. In the presented trial sequence, the signal contrast selected for each trial is represented by the dot's position on the ordinate, and the trial's response (*Interval 1 or Interval 2*) is represented by the dot's color (black, blue). The correctness of responses in the trial sequence can be inferred from dot location and color as follows: correct responses are marked by *blue dots above* and *black dots below* the 0 position of the abcissa's relative contrast scale; black dots above and blue dots below mark incorrect responses. The 1-D marginal pdfs for the sensitivity and decision parameters are presented in the lower panels. The stimulus search is calculated over the one-dimensional space of relative signal contrast. Therefore, unlike previous FC methods, the method selects both the signal contrast and the interval in which it's presented. This strategy is needed to estimate the decision criterion (interval bias).

## Appendix A

### Justifying the *d*′ psychometric function (contrast transducer function)

In the spatial vision literature, there have been many approaches to parameterizing the contrast transducer function (Legge and Foley, 1980; Wilson, 1980; Foley and Legge, 1981; Smith et al., 2010). The choice of our parameterization (Equation 1) is based on a wealth of data from studies on noisy observer models (see Lu and Dosher, 2008). To further examine the validity of this function, we collected additional data for a simple detection task using the MCS. The goal of the *MCS* sampling scheme was to sample the dynamic region of the empirical psychometric function, and at least 2 points each from its lower and upper asymptotes (Wichmann and Hill, 2001b; Garcia Perez and Quintana). For each session, 50 trials were collected at each of eight stimulus levels (400 total per session). Eight observers, who each completed a range of 1 to 13 sessions, completed a total of 18,000 trials. The resulting data were fit with the same model of the empirical psychometric function for simple detection:

(A1) $$\Psi_{yes}(c)=1-G\left(\lambda -d^{\prime }(c)\right),$$

with different functional forms of the *d*′ function. In addition to the function applied in this study:

(A2) $$d^{\prime }(c;\tau ,\gamma ,\beta )=\frac{\beta {\left(c/\tau \right)}^{\gamma }}{\sqrt{(\beta^{2}-1)+{\left(c/\tau \right)}^{2\gamma }}},$$

and a common power-law model of sensitivity (Foley and Legge, 1981; Kontsevich & Tyler, 1999; Klein, 2001):

(A3) $$d^{\prime }(c;\alpha ,\beta )={\left(c/\alpha \right)}^{\beta }.$$

We fit other functional forms from the spatial vision literature:

$$d^{\prime }(c;\alpha ,\beta )=\frac{\beta c^{2.4}}{c^{2}+\alpha^{2}}\;$$

                                                       (A4; Legge and Foley, 1980)

d′(c;α,β,γ)=α((1+cγ)13−1)(213−1)(β+c).8 

                                                       (A5; Wilson, 1980)

d′(c;α,β,γ)=β(1−e(Cα)γ) .

                                                       (A6; Smith et al, 2010)

This list of functions represents the range of approaches to fitting sensitivity psychometric functions in detection tasks, but is not exhaustive. Further, to test the validity of the *qYN*'s simplifying assumption that fixed the upper asymptote parameter, β, and the potential validity of a model with a strict prior on psychometric steepness, γ, we also fit the *d*′ function defined in Equation (1) under conditions fixing only β (β = 5) or both β and γ (β = 5 and γ = 2).

The result is a total of 7 model conditions: three correspond to conditions for fitting our preferred function (Equation A2), under different levels of parameter constraints, and four correspond to *d*′ functions obtained from the literature (Equations A3–6). Data from each of 45 testing sessions (400 trials) were fit independently using χ^2^ minimization (see Appendix B). To evaluate the goodness-of-fit (*r*^2^), the mean and standard deviations (in %) for the functions defined in Equations (A2–6) were: [1] *98.4* (*1.60*), [2] *97.4* (*1.76*), [3] *98.0* (*1.52*), [4] *97.7*(*1.61*), and [5] *98.3* (*1.59*). Furthermore, constraining the fits of the *d*′ function used for the *qYN* reduced the goodness-of-fit only slightly, for [6] 97.8 (1.54) for fixed β, and [7] 97.0 (2.23) for fixed β and γ.

The equivalent empirical fits provided by different forms of the sensitivity function suggest that investigators can apply Bayesian adaptive inference to estimate the functional forms of the sensitivity function that they prefer. Furthermore, the equivalent fits provided by the constrained *qYN* models suggest that convergence of the *qYN* can be improved by placing a strict prior on the parameter defining psychometric steepness. This interesting possibility requires more investigation and psychophysical validation.

## Appendix b

### Fitting the SDT model to MCS data

For fitting the MCS model, it was assumed that the upper asymptote of the sensitivity function was 5.0 and that the observer's lapse rate was 1%. Best fits were obtained using χ ^2^ minimization between observed and fitted response probabilities. The χ ^2^ score was calculated by:

(A4) $$\chi^{2}=\sum \frac{{\left(p_{observed}-p_{fitted}\right)}^{2}}{[p_{fitted}\times (1-p_{fitted})]\;/n},$$

where *n* is the number of trials collected at each stimulus level. It's been noted that standard χ ^2^ tables are often inappropriate for testing goodness-of-fit for psychophysical data (Klein, 2001; Wichmann and Hill, 2001a). Therefore, we generated bootstrapped χ ^2^ distributions (Maloney, 1990; Wichmann and Hill, 2001b) by using the original fitted parameters, θ_MCS_ to generate bootstrapped datasets (across all four tasks) via Monte Carlo simulations (10,000 iterations). Fitting the same model to each re-sampled data set provided re-fitted probabilities, which were used to calculate χ ^2^ scores, relative to the original fitted probabilities:

(A5) $$\chi^{2}=\sum \frac{{\left(p_{resampled}-p_{fitted}\right)}^{2}}{[p_{fitted}\times (1-p_{fitted})]\;n},$$

where *n* = 8 + 16 + 8 + 11 = 43 stimulus levels used in the *MCS* trials. This produced a bootstrapped distribution of χ^2^ differences between fitted and re-sampled probabilities. The empirically observed chi-score, χ^2^_*observed*_, defined by the initial fitted and observed probabilities, was compared to the bootstrapped distribution via a percentile test; this comparison yielded a percentile score (*p*-value) via the percentage of resampled scores, χ^2^_*resampled*_, exceeding the empirical chi-score.
